# NEWS

**DOI:** 10.7189/jogh.03.020202

**Published:** 2013-12

## THE BILL AND MELINDA GATES FOUNDATION

• Bill Gates clashed with Dambisa Moyo, a prestigious Zambian economist over the value of development aid in tackling poverty. In her book “Dead Aid”, Ms Moyo argues that aid has actually increased poverty, corruption and dependency in Africa, while Mr Gates has commented that books like hers are “promoting evil”. The clash has been prominent over Twitter, mostly light–heartedly. The complex aid debate continues on and is becoming increasingly polarised, highlighting an apparent difficulty in quantifying impact of aid provisions. (*The Guardian,* 31 May 2013)

• The European Commissioner for Research and Innovation signed an agreement with the Bill and Melinda Gates Foundation, with both parties pledging to fund research into drugs, vaccines and diagnostics to combat HIV, tuberculosis, malaria and other neglected infectious diseases. Currently, these poverty–related diseases affect more than one billion people worldwide, many of whom are living in developing countries where there is little or no access to safe and affordable treatment. Despite cuts in other areas of the EU budget, the Commissioner’s research and innovation directorate budget has remained intact; and this agreement is part of the EU Horizon 2020 programme. It is anticipated that it will allow the development of at least one new and better health product per year. Mr Gates described the partnership as “critical to the success of our common mission…we can together improve the lives of millions.” (*EUACTIV,* 10 June 2013)

• The Foundation announced its latest Grand Challenges Explorations, a programme to encourage bold approaches to improving the lives of the world’s poorest people. Grant in the amount of US$ 100 000 are available for new ideas on: how to encourage the world’s poorest people to seek health care; developing a new condom; and reducing diarrhoea, one of the world’s biggest killers of children. Projects demonstrating potential could receive additional funding up to US$ 1 million. The simple online application is open to anyone from any discipline (including cross–disciplinary applications) or organisation. Since its launch in 2008, it has funded more than 850 grants in 50 countries. (*BMGF*, 5 Sept 2013)

• The Brazilian health minister announced a partnership between Bio–Manguinhos (Brazil’s leading medical research facility) and the Bill and Melinda Gates Foundation to develop and export an affordable combined measles and rubella vaccine to low income countries. Measles is a leading cause of children under five in these countries, and rubella has serious consequences for pregnant women and their babies. Brazil follows in the footsteps of China and India, whereby major emerging markets develop low–cost drugs for lower–income nations. The combined vaccine currently has limited availability, and Bio–Manguinhos will increase availability by 30 million doses annually, at much lower costs than present. The Gates Foundation has granted US$ 1.1 million to support clinical trials and the vaccine is expected to reach the market by 2017. (*Reuters*, 28 Oct 2013)

• The Foundation announced a new initiative to develop vaccines to tackle child mortality in the developing world, where preventable diseases such as malaria and pneumonia are a leading cause of death. This scheme, known as the Vaccine Discovery Partnership, will see the Foundation working alongside pharmaceutical companies. Sanofi and GlaxoSmithKline (GSK) are the first to sign up, and it is hoped that others will soon follow. A project with GSK is already under way, on building thermostability into vaccines to enable their safer delivery in countries with limited resources. By working with pharmaceutical companies the Foundation hopes to reduce the costs and risks associated with early–stage vaccine research, saying that “this will be a win for everyone involved but most importantly for the children around the world who will get the life–saving vaccines they need.” (*BMGF*, 29 Oct 2013)

## THE GAVI ALLIANCE

• The non–profit International Medica Foundation has developed the first rotavirus vaccine suitable for newborn babies. The vaccine–preventable rotavirus can cause life–threatening diarrhoea. In developing countries, it is more common amongst babies and younger children compared to developed countries. In China alone, it causes 4900 deaths and 329 000 hospitalisations of children and infants, causing more than half a million deaths worldwide. Existing rotavirus vaccinations are expensive to produce, transport and store, carry higher risks of intestinal blockages, and are unsuitable for newborn babies. The new vaccination overcomes these problems, and is suitable for global use. They have been sub–licensed to Shanghai BravoBio Co. Ltd of China, a private biotechnology company based in Shanghai, for use in China. The Foundation is now seeking more private sector partners to extend global coverage. (*WSJ*, 27 Jun 2013)

• A major initiative to eliminate measles outbreaks in the DR Congo started in September 2013. The aim is to vaccinate 6.8 million children aged from six months to nine years in two of the country’s provinces, and to give two drops of polio vaccine to children under five. This will be rolled out to other parts of the country by 2014. Thanks to concerted efforts, polio has not occurred in the country since December 2011, but measles outbreaks continue and are a leading cause of childhood deaths despite the existence of effective vaccinations. As measles spreads very readily, high rates of coverage are needed to provide protection. Ongoing measles outbreaks show that routine vaccination coverage in the DR Congo is too low. (*GAVI Alliance*, 24 Sept 2013)

• GAVI is on track to meet its ambitious targets of supporting developing countries to immunise an additional quarter of a billion children by 2015, thus preventing nearly four million deaths. Its report found that the gap in access to immunisation between low– and high–income countries is closing, and that GAVI is on target to help avert nearly four million future deaths. GAVI has made significant progress towards achieving its four strategic goals of: accelerating the uptake of under–used and new vaccines; strengthening health systems to improve coverage; improving long–term predictability and stability of financing; and helping to improve vaccine market conditions for developing countries. “Vaccines are already widely recognised as one of the most cost–effective public health tools, but we cannot rest until all children, regardless of where they live, have access to the best possible protection against vaccine–preventable diseases,” says Dr Seth Berkley, CEO. (*GAVI Alliance*, 14 Oct 2013)

• An advisory group to the Indian government approved the national scale–up of the pentavalent vaccine. The extension will be accompanied by careful monitoring for any adverse reactions to the vaccine. The “five–in–one” pentavalent vaccine combines vaccinations for diphtheria, tetanus, whooping cough, hepatitis B and *Haemophilus influenza* type b (Hib, causing meningitis, pneumonia and otitis). It will reduce the number of injections needed from nine to three. Globally, Hib is the second most common cause of bacterial pneumonia deaths, and the third biggest vaccine–preventable death in children aged under five. Each year, it causes eight million serious illnesses, and claims 400 000 lives. In welcoming India’s decision, the GAVI Alliance said that it could save one million lives by 2020. (*GAVI Alliance*, 30 Oct 2013)

• Globally, pneumonia remains the single biggest killer of children aged under five, with over a million deaths each year. There is a range of tools against pneumonia, making it a highly preventable disease. To deal with the challenge, the GAVI Alliance will support the introduction of pneumococcal vaccine across 50 countries by 2015. “The GAVI Alliance is helping to accelerate the fight against pneumonia by increasing access to pneumococcal vaccines, thanks to GAVI’s innovative Advance Market Commitment, but also to the five–in–one pentavalent vaccine which protects against Haemophilus influenza type b, another major cause of pneumonia,” said Mr Seth Berkley, CEO. (*GAVI Alliance*, 12 Nov 2013)

## THE WORLD BANK

• Jim Yong Kim, President of the World Bank, has called for a global ‘science of delivery’ to ensure that development aid is delivered consistently and its benefits are widespread. He believes that a systematic approach would greatly improve outcomes, and the four main components of this new science would be: knowledge collection to support frontline implementation, teaching of delivery skills based on the experience of the most successful practitioners, incorporation of research to encourage innovation and development of frameworks to adapt and explain successful solutions to delivery problems. (*McKinsey on Society*, 28 Apr 2013)

• The World Bank’s *Doing Business* annual survey measures the costs to companies of business regulations in each of the 183 countries surveyed. By supporting transparency and spurring improvements in business environments, it aims to promote private sector development and wealth creation within each country – crucially important to developing countries. It is also one of the lending criteria for low–income countries. Yet there is controversy over the Bank’s methodology and its focus on de–regulation. There are real concerns that some countries try to improve their rankings by diluting vital safety and environmental protection legislation. This criticism has come from some developing countries (including China), aid agencies and trade unions. This prompted a review of the report in June 2013, which recommends overhauling the indicators, and reviewing its economic rationale to ensure fairness. (*The Guardian*, 31 May 2013)

• At the London Nutrition for Growth Summit in June 2013, global leaders committed to giving Ł 3 billion in funding to tackle the problem of malnutrition in the world’s poorest countries. The summit was primarily supported by The Children’s Investment Fund Foundation and consisted of both private and public sector agencies, including the World Bank. Research by Save the Children identifies lack of nutrients in the first 1000 days of life as a major risk factor for learning difficulties, greatly stunting economic and social development within emerging countries. The charity’s chief executive Justin Forsyth highlighted the importance of the summit in recognising this significant danger of malnutrition and urges them to “commit to tackle its scourge for good.” This increase in funding is therefore an impressive start to the G8 period. (*Financial Times,* 8 Jun 2013)

• Cash transfers are increasingly used to alleviate poverty and support development in emerging economies. The idea is simple: instead of spending cash on schools and infrastructure, individuals or businesses directly receive a fixed cash amount. There are two types of transfer: conditional (CCT) and unconditional (UCT). UCT recipients chose how to use the money, and CCT recipients have conditions attached, eg, school attendance, vaccinations or business planning. The World Bank’s assessment shows that both are highly effective in pulling people out of poverty. Most UCT recipients spent the money on projects that raise their income. They are invaluable when the main cause of poverty is shortage of capital and are very cost–effective. But by attaching conditions such as school or clinic attendance, CCT is better for long–term development where complex factors (eg, low literacy levels, poor child health and nutrition) keep people in poverty over time. (*The Economist*, 26 Oct 2013)

• The Zambian Minister for Tourism and Arts, Ms Sylvia Masebo, launched the World Bank *Tourism in Africa* report. It provides strategies to maximise the economic power of tourism, in order to boost growth and improve livelihoods. In 2012, sub–Saharan Africa attracted 33.8 million visitors, compared to 6.7 million in 1990. Income from tourism amounted to US$ 36 billion, contributing 2.8% to the region’s GDP and creating 5.5 million direct jobs. The report notes that there is huge untapped potential for tourism in sub–Saharan Africa, which has expansive beaches, plentiful wildlife, and nature, culture and adventure opportunities. Domestic tourism is also expected to increase as disposable incomes rise. (*World Bank*, 13 Nov 2013)

## UNITED NATIONS

• Between 1990 and 2010, the proportion of the world’s population living on less than US$ 1.25 a day (ie, extreme poverty) halved to 21%, or 1.2 billion people. This means that the Millennium Development Goal (MDG) of halving poverty rates by 2015 has already been met. This success has led to commitments to eradicating extreme poverty by 2030, endorsed by President Barack Obama. The British Prime Minister, David Cameron, chaired a UN panel that set out a blueprint for eradicate extreme poverty, and proposals to improve access to food and water, better governance, boosting jobs and economic growth, and strengthening human rights. It states the role of climate change in meeting this goal, as it affects poorer people disproportionately. Whilst welcoming the “zero goal” of ending extreme poverty, some aid agencies called for more emphasis on reducing inequalities, which are not specifically addressed in the report. (*The Guardian*, 30 May 2013)

• UN Secretary General Ban Ki–moon appealed to philanthropists to “make a smart investment in the world’s future” by joining UN efforts to accelerate the fight against five of the most deadly infectious diseases (malaria, polio, tetanus, measles and HIV) which kill millions every year. Significant progress has been made in fighting these diseases, and Mr Ki–moon declared that it is possible to eliminate them within five years. He called on the private sector and philanthropists to join the fight, saying that every US$ 1 invested in fighting malaria in Africa, US$ 40 is generated in gross domestic product, strengthening economies and peoples’ livelihoods. (*UN News*, 5 Jun 2013)

• The UN World Investment Report 2013 reveals that foreign direct investment (FDI) inflows to the world’s least developed countries rose by 20% in 2012, reaching a record US$ 26 billion. For the first time, least developed countries overtook developed countries as the main FDI recipients. The increase was led by Cambodia, the Democratic Republic of the Congo, Liberia, Mauritiana, Mozambique, Liberia and Uganda. More of this investment originated in developing countries themselves, notably India, which invested in diverse projects across Africa and Asia. The UN Secretary General, Mr Ban Ki–moon, called this a “source of reflection and inspiration.” He re–iterated the linkages between FDI and development. (*UN News Centre*, 27 Jun 2013)

• In September the UN launched an aid appeal to support farmers and herders in the Sahel region of Africa. Stretching from the Red Sea to the Atlantic Ocean, encompassing areas between the Sahara desert in the north and the Sudanese savannah to the south, Sahel suffers from political instabilities, security issues and human rights violations. These factors, combined with increasing grain prices due to the 2012 drought, have left 11 million people at risk of hunger. Mr Robert Piper, the region’s UN Regional Humanitarian Coordinator, asked the UN for US$ 1.7 billion, but only 36% of it has been received. The appeal has been re–launched to attempt to secure funding ahead of the next agricultural season. (*UN News Centre*, 4 Sep 2013)

• The IMF announced a reduction in its forecasts for global economic growth for 2013 and 2014. The revised figure shows an expected global growth rate of 2.9% for 2013 (down by 0.3%), and 3.6% for 2014 (down by 0.2%). The main reasons are weakness in emerging economies, despite relatively strong growth in developed economies such as the USA and UK, and stabilisation in the Eurozone. Slower rates of economic growth in countries such as Brazil, China and India are holding back global economic growth. This is partly caused by the impact of the US debt crisis, and fears that it could reduce economic stimulation measures as a result. These fears have already affected interest rates and the cost of borrowing in emerging economies, thus dampened their growth prospects with knock–on effects on poverty reduction. (*BBC News*, 8 Oct 2013)

## UN AIDS

• The UN AIDS Update on Africa charts the African AIDS response. It shows a huge increase in the number of people receiving antiretroviral treatment for HIV, from less than 1 million in 2005 to 7.1 million in 2012. Sixteen African countries are ensuring that at least 75% of pregnant women living with HIV receive antiretroviral treatment, lessening transmissions to babies. This has led to a 32% drop in AIDS–related deaths between 2005 and 2011. However, Africa remains the continent most affected by HIV. In 2011 there were 1.8 million new HIV infections, and 1.2 million people died of AIDS–related illnesses. (*UN News Centre*, 21 May 2013)

• With more than 34 million people infected, HIV is a major contributor to the disease burden in many countries. Globally, the management, control and policy of this epidemic are instrumental in reshaping conventional wisdom in public health, research and practice. Through innovative approaches to prevention and treatment programmes, it has influenced cultural attitudes and social behaviours, and bridged traditional boundaries between public health and clinical medicine. With its human rights approach to health, HIV/AIDS has addressed crucial issues on the right to treatment, and affordable access to vital medicines. HIV/AIDS has attracted private philanthropy such as the Bill and Melinda Gates Foundation, leading to new models of public health practice through Public–Private Partnerships, funding scientific investigations, global health initiatives, and building crucial health care delivery infrastructure in developing countries. Thus, HIV/AIDS has replaced traditional approaches of “international health” by inventing innovative approaches to “global health”. (*New England Journal of Medicine*, 6 Jun 2013)

• On 20 June 2013, the US Supreme Court struck down section 7631(f) of the Leadership Act. This section meant that no funds made available under the Leadership Act may “provide assistance to any group or organisation that does not have a policy explicitly opposing prostitution and sex trafficking.” The Court held that the provision violated the First Amendment of the US Constitution which protects free speech. It means that AIDS groups can work more effectively with sex workers, who have much higher risks of HIV infection than the general population. The USA is a major sponsor in the fight against HIV and AIDS, so this greatly strengthens the global HIV response. (*UN AIDS*, 21 Jun 2013)

• WHO recommends widespread circumcision as an effective additional intervention in regions with HIV epidemics. It may reduce the risk of heterosexual male infection by approximately 60%, and the WHO has a target of 20 million circumcisions in Africa by 2015. It approved the use of a circumcision device, PrePex^TM^ that incorporates a band that puts pressure around the foreskin, causing tissue death and circumcision. The device paves the way for more non–surgical circumcisions, which is potentially safer, simpler and cheaper. However, circumcision does not eliminate HIV infection or prevent transmission, so wider measures (eg, condom usage) are still crucial. (*WHO*, 31 October 2013)

• New figures from UN AIDS reveals that out of a total of 35.3 million people living with HIV, an estimated 3.6 million are aged 50 years or over. The majority of these are in low and middle income countries. UN AIDS is concerned that this group’s specific health needs are being missed, leading to lives being lost. This “aging” is due to the success of antiretroviral treatments. UN AIDS highlight that HIV prevention and testing needs to be tailored to the needs of this group, the importance of timely drug therapy as the immune system weakens over time, and integrating HIV services into other health screening services aimed at this age group. (*UN AIDS*, 1 Nov 2013)

## UNICEF

• Some of the world’s poorest countries have cut maternal and child mortality rates by 50% or more since 2000, according to the UNICEF report *Accounting for Maternal, Newborn and Child Survival,* although progress has been lagging in others. The report assesses progress in the 75 countries that account for more than 95% of all maternal and child deaths. Rwanda, Botswana, and Cambodia have made notable progress in reducing mortality since 2000. The key findings are: a significant drop from 543 000 in 1990 to 287 000 in the number of women dying each year from pregnancy– or childbirth–related complications; and deaths among children under five years dropped from 12 million in 1990 to 6.9 million in 2011. (*UNICEF*, 27 May 2013)

• A Save the Children Report states that childhood under–nutrition could have considerable effects on literacy and learning, with serious implications for later life. A new study interviewed and tested over 7000 children at key points in their lives, and showed that malnutrition in early life is associated with poorer educational performance, with undernourished children performing worse in mathematics, reading and writing tests and being 19% less likely to be able to read a simple sentence when aged eight. The scale of this problem is potentially enormous; currently one in four of all children under 5 are stunted due to under–nutrition. The research was backed publicly by a group of children’s authors, who are calling on G8 Governments to increase their response to tackle hunger worldwide. (*Save the Children*, 27 May 2013)

• With the fresh challenges of the post–2015 era fast approaching, the deficit of data in global health research must be urgently addressed. This “data revolution” is vital in addressing the problems of marginalised groups to “leave no one behind,” one of the key promises in the UN High–Level Panel Report on Eminent Persons. The report criticises the Millennium Development Goals for insufficiently focusing on the most excluded people, particularly those living with disabilities, or outside traditional household units. Despite the emergence of new technology for data collection, the household survey is recommended as an efficient, cheap and comprehensive method for broadening coverage and collecting better information about groups particularly affected by social inequality. Household surveys ensure that measurements and monitoring of groups previously sidelined in data collection are integrated into well–established survey systems. (*The Guardian,* 10 Jun 2013)

• UNICEF published its annual assessment of developing and strengthening supply chains, which improves access to life–saving supplies for children and their families. Optimising UNICEF’s supply chains supports the realisation of children’s rights to health, education, nutrition and protection, and ensures that UNICEF’s programmes have a higher impact. The recent implementation of changes to its supply planning and accountancy systems have led to savings of 4% in supplies procured. UNICEF reported that the new systems also have clear advantages in terms of efficiency, visibility and oversight. These gains will become more evident as the new systems and techniques stabilise and mature. (*UNICEF*, 30 Jun 2013)

• UNICEF reinforced its commitment to transparency and accountability by publishing its Information Disclosure Policy. It outlines UNICEF’s commitment to sharing information on its programmes and operations with everyone. From September 2012 onwards, UNICEF also had full public disclosure of all its internal audit reports, and annual programme reports. As a signatory to the International Aid Transparency Initiative, UNICEF will publically disclose information on aid spending. This ensures that users can easily find, use and compare data. Information will be available in user–friendly formats. Ultimately, information disclosure makes agencies more efficient, responsible, collaborative and better able to deliver their goals. (*UNICEF*, 24 Oct 2013)

## WORLD HEALTH ORGANIZATION

• The world is unprepared for a massive virus outbreak, warned Dr Keiji Fukuda of the WHO, amid fears that the H7N9 bird flu virus striking China could mutate into a form that spreads easily amongst people. According to the latest data, 135 people in China have been infected, including 44 deaths. It is one of an array of avian flu viruses, most of which pose little or no risk to humans. The more common strain of avian flu, H5N1, has killed more than 360 people globally. The year 2003 saw the emergence of the SARS virus and the re–emergence of the H5N1 virus. WHO acknowledged that any new virus that infects humans could become a global health threat. Rapid–reaction systems are crucial, as health authorities are hampered by lack of knowledge about such diseases, and how it can spread amongst people. (*AFP*, 21 May 2013)

• The 66th World Health Assembly (WHA) concluded with agreement on a range of new public health measures and recommendations aimed at securing greater health benefits for all people, everywhere. During her closing address, the WHO Director–General Dr Margaret Chan issued a warned over the novel coronavirus, citing lack of understanding and its threat to the entire world. WHO has already recognized that coronavirus is an important and major challenge for all affected countries, as well as the rest of the world. It calls for understanding how people are getting infected, and identifying the main risk factors for either infection or development of severe disease, as a matter of urgency. (*WHO*, 27 May 2013)

• Current models for developing new medical and pharmaceutical products are under review, and new models are needed to meet changing market requirements in developing and developed countries. This theme was addressed at the joint symposia on changing business models between the WHO, World Trade Organisation and World Intellectual Property Organisation. Participants included GAVI Alliance, South African Medical Research Council, and the Bill and Melinda Gates Foundation. The WHO, WIPO and WTO are also expanding their capacities on the interface between intellectual property, trade and public health by strengthening their collaboration. This is part of wider efforts to improve access to medicines by the world’s poor, including new and more effective medicines. (*WTO*, 30 Jul 2013)

• The WHO report on global tuberculosis (TB) control reveals that in 2012, the number of people with TB fell to 8.6 million people, and deaths to 1.3 million, with treatment saving 22 million lives. The world is on track to meet the Millennium Development Goal of a 50% reduction in the mortality rate by 2015, and reversing TB incidence. To keep up this momentum, WHO highlighted that more must be done to reach the 3 million TB patients “missed” by health systems, and for faster responses to drug–resistant TB. More funding is essential to meeting the challenges in scaling–up screening and access to treatment. (*WHO*, 23 Oct 2013)

• In October 2013, the WHO published its annual World Health Statistics, which reports progress toward the Millennium Development Goals. Recently, progress has accelerated in many countries, but large gaps remain both between and within countries. Twenty seven countries have met the MDG target of a two–thirds reduction in under–five mortality rates. However, the overall rate of decline is not enough to meet this target globally by 2015. Under–nutrition is the underlying cause of death in 35% of cases. The decline is under–nutrition is on track to meet the MDG, but wide variations persist. There has been a substantial reduction in global maternal mortality rates, but the rate of decline needs to double if the target is to be met. The MDG of halving the proportion of the world’s population without access to safe drinking water was reached in 2010, although some regions will miss this target and there are sharp discrepancies in access between richer and poorer households. Progress is slower towards the sanitation goal, with more than 2.5 billion people worldwide lacking access to better sanitation. Access to medicines is still problematic in low and middle–income countries, with an average of only 57% of generic medicines being readily accessible through the public sector. (*WHO*, 31 Oct 2013)

